# Comment on “Invisible Victims: Delayed Onset Depression among Adults with Same-Sex Parents”

**DOI:** 10.1155/2016/3185067

**Published:** 2016-12-19

**Authors:** Nathaniel Frank

**Affiliations:** What We Know Project, Columbia Law School, New York, NY, USA

I was appalled, if not surprised, to see the publication of Donald Sullins' study, “Invisible Victims: Delayed Onset Depression among Adults with Same-Sex Parents” in* Depression Research and Treatment* (2016) [1].

Sullins claims that having same-sex parents increases the likelihood of suffering from depression, abuse, parental distance, and obesity and concludes that households with gay or lesbian parents “may be problematic or dangerous” for the “dignity and security” of their offspring. Yet to support these conclusions, Sullins would have needed to compare same-sex- and different-sex-headed households in which it is known that no family disruptions occurred (or that the same level of such disruptions occurred in each group).

Instead, he draws sweeping, outlier conclusions (74 studies collected by my research team at Columbia Law School's What We Know Project [2], which aggregates scholarship with public policy implications, have found that parent sexual orientation does not affect the wellbeing of children) that can only be reached by fudging the way gay- or lesbian-headed households are discussed and compared to households headed by heterosexuals.

Sullins achieves this through a crucial elision between households in which a child spent some time in a home headed by a same-sex couple and families in which a child was actually raised, from birth, by a stable same-sex couple, a situation more auspicious for healthy child development. This conflation of household stability with parent gender fatally mars his conclusions, which are much more damning of gay and lesbian parenting than are warranted by his data.

Sullins claims that his study examines “children raised by same-sex parents into early adulthood.” But in fact, he has zero basis to draw this conclusion, as he is applying a wholly untenable definition of “raised by.” All he knows about his dataset is that his subjects, who ranged in age from 12 to 18, spent some of their teenage years with a parent who at some point had a same-sex partner. Since we do not know if that partner was ever actually a parent, legally or otherwise, it is inaccurate to characterize such households as “same-sex parented” as Sullins does eleven times. It is even more inaccurate to claim that those living in these households were “raised by” same-sex parents, since we know nothing about the youths' parentage before their teenage years.

Not only is there no basis to conclude that these subjects were raised by same-sex parents, but also there is every reason to believe they likely were not. This is made clear by comparing the number of same-sex couples with children to the number of gay or lesbian parents overall. Census and scholarly data show that about a quarter million Americans are currently parenting as part of a same-sex couple [3]. Around 139 million Americans are aged 18–50 [4], of whom 3.5% are LGB [5] and 35% are raising a child [3]. An estimated 1.7 million gay or lesbian Americans are currently parents; that is, they are parenting but not as part of a same-sex couple. That means that only about 15 percent of households with at least one gay parent are ones in which a same-sex* couple* is parenting, let alone has raised a child from birth, a higher bar that only applies to households in which the parents have stayed together over time and are known to both be parents to the child(ren).

This descriptor, of course, is the key variable in the discourse on optimal child-rearing because of the well-established fact that children who experience divorce or other family disruptions are at higher risk for a number of disadvantages, including the ones that Sullins inaccurately associates with “same-sex parented” households.

What Sullins has done makes no more sense than surveying a hospital to derive mortality rates. It is hard to imagine that Sullins does not know this and equally hard to watch his misleading findings get past peer review.
